# Recurrent severe infections in a child with *STAT3*-associated hyper-IgE syndrome

**DOI:** 10.1016/j.jdcr.2025.09.050

**Published:** 2025-10-27

**Authors:** Nadia Nur Asifa, Paranita Ferronika, Dyah Ayu Mira Oktarina, Afrilia Intan Pratiwi, Sumadiono, Cahya Dewi Satria, Retno Danarti, Tuntas Rayinda

**Affiliations:** a Department of Dermatology and Venereology, Faculty of Medicine, Public Health and Nursing, Universitas Gadjah Mada, Yogyakarta, Indonesia; b Department of Dermatology and Venereology, Sardjito Hospital, Yogyakarta, Indonesia; c Department of Anatomical Pathology, Faculty of Medicine, Public Health and Nursing, Universitas Gadjah Mada, Yogyakarta, Indonesia; d Department of Anatomical Pathology, Sardjito Hospital, Yogyakarta, Indonesia; e Department of Child Health, Faculty of Medicine, Public Health and Nursing, Universitas Gadjah Mada/ Sardjito Hospital, Yogyakarta, Indonesia; f Department of Child Health, Sardjito Hospital, Yogyakarta, Indonesia

**Keywords:** hyper-IgE syndrome, Staphylococcal infections, STAT3 transcription factor.

## Case description

A 15-year-old Javanese boy presented with painless, progressively enlarging neck lumps over the past month. He had recurrent skin abscesses since early childhood, requiring multiple surgical drainages, and had generalized xerosis and eczematous rashes since infancy. He had a history of severe appendicitis with peritonitis and sepsis, pulmonary tuberculosis, and pneumonia. There was no family history of similar conditions or consanguinity.

**Fig 1:**
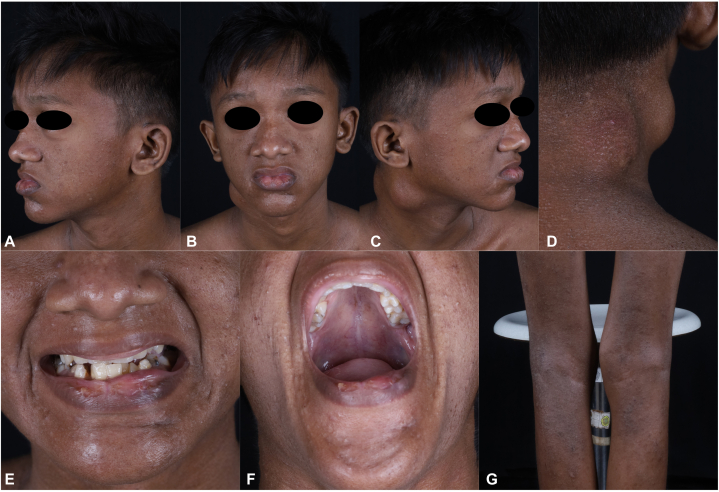
**A-C,** Dysmorphic facial features, **(D)** cold abscess on the neck, **(E)** prognathism, **(F)** high-arched palate, and **(G)** eczematous lesions and xerotic skin.

The patient was underweight (body mass index: 13.85 kg/m^2^). Dermatologic examination showed 2 fluctuant, mobile, non-tender cystic nodules measuring 3 cm and 5 cm on the right side of the neck, generalized xerosis, hyperpigmented papules and plaques in flexural areas, and excoriations. Distinctive craniofacial features were observed, including deep-set eyes, increased intercanthal distance, broad nasal bridge, prognathism, and a high-arched palate (Fig 1).

**Fig 2:**
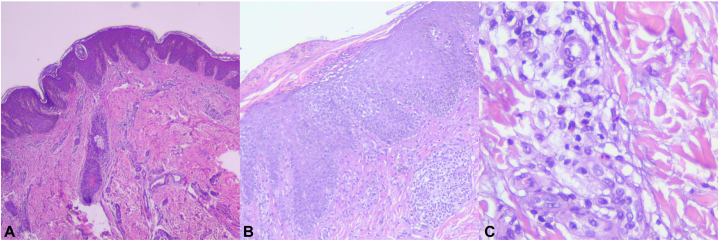
Histopathologica picture of skin lesion in cubital fossa showed **(A)** irregular acanthosis (hematoxylin-eosin (HE), 40×), **(B)** focal parakeratosis and moderate spongiosis (HE, 100×), **(C)** perivascular infiltration of eosinophils, lymphocytes, and plasma cells (HE, 400×).

**Fig 3:**
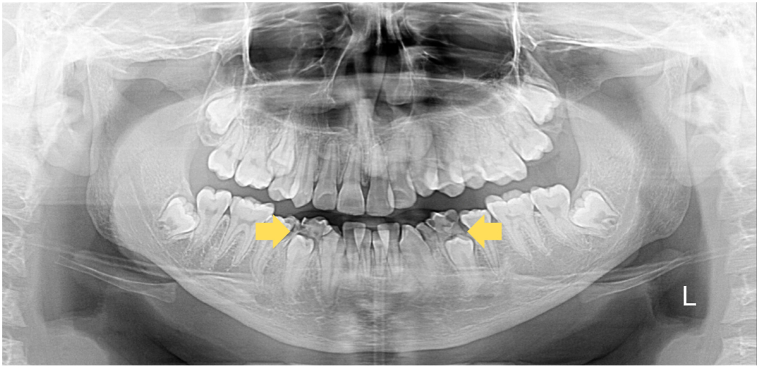
Dental panoramic X-ray showed retention of both mandibular first premolar primary teeth (*yellow arrows*).

Blood tests revealed anemia (hemoglobin 10.7 g/dL), leukocytosis (12,300/μL) with eosinophilia (602.7/μL), thrombocytosis (766,000/μL), and markedly elevated serum immunoglobulin E (IgE, 5909 IU/mL). Skin biopsy from the cubital fossa showed subacute spongiotic dermatitis with eosinophilic infiltration (Fig 2). Panoramic dental radiography demonstrated retained mandibular primary teeth (Fig 3). Bacterial culture from a drained abscess yielded *methicillin-resistant Staphylococcus aureus*. The patient scored 32.47 on the National Institutes of Health hyper-immunoglobulin E syndrome (HIES) scoring system, above the 30-point threshold suggesting probable HIES. Whole-exome sequencing of the proband’s venous blood DNA revealed a heterozygous STAT3 missense variant (NM_139276.3:c.1909G>A; p.Val637Met), confirming HIES.

**Question: Which of the following is a classic triad of symptoms in HIES?**

**A.** Recurrent fungal infections, asthma, high IgE
**B.** Recurrent bacterial infections, severe atopic dermatitis, and high IgE
**C.** Atopic diathesis, trichorrexis invaginata, and ichtyosiform erythroderma
**D.** Atopic diathesis, skeletal anomalies, and eosinophilia
**E.** Dry skin, recurrent pneumonia, and high IgE

Correct answer: **B.**

## Discussion

Hyper-immunoglobulin E syndrome (HIES, Online Mendelian Inheritance in Man #147060) is a rare primary immunodeficiency characterized by triad of recurrent bacterial infections, severe atopic dermatitis, and markedly elevated serum IgE.^1^ Associated features include characteristic facies, musculoskeletal anomalies, and retained primary teeth.^2^ HIES may arise from de novo mutations or be inherited in either an autosomal dominant or autosomal recessive pattern. The autosomal dominant form is most commonly associated with pathogenic variants in *STAT3* that impair Th17/IL-1, predisposing to *Staphylococcus aureus* and *Candida* infections, with dysregulated IL-6/IL-10 contributing to eczema-like lesions.^2^

Diagnosing HIES can be challenging since its features overlap with common conditions like eczema and recurrent infections.^2^ The characteristic triad of atopic dermatitis, recurrent bacterial infections, particularly skin and pulmonary infections, and significantly high serum IgE levels should be recognized prior to genetic testing, which remains a gold standard.^3^ Serum IgE testing, though not routine in all atopic eczema patients, should be considered in cases with severe recurrent infections, systemic features suggesting inborn errors of immunity, or characteristic facies.^4^ The National Institutes of Health scoring system, with scores >30, has good sensitivity (87.5%) and specificity (80.6%) for predicting *STAT3* mutations in patients with IgE >1000 IU/mL.^3^

This case highlights the risk of opportunistic infections in HIES, exemplified by recurrent multifocal severe infections, and underscores the importance of timely diagnosis to enable appropriate management to prevent complications such as bronchiectasis, skeletal abnormalities, and growth delay.^5^

## Conflicts of interest

None disclosed.
